# The mediating role of coping styles in the relationship between second victim experience and professional quality of life among nurses: a cross-sectional study

**DOI:** 10.1186/s12912-023-01473-9

**Published:** 2023-09-12

**Authors:** Xizhao Li, Chong Chin Che, Yamin Li, Ling Wang, Mei Chan Chong

**Affiliations:** 1 Department of Nursing Science, Faculty of Medicine, University of Malaya, 50603 Kuala Lumpur, Malaysia; 2 Clinical Nursing Teaching and Research Section, The Second Xiangya Hospital, Central South University, Changsha, China; 3 Florence Nightingale Faculty of Nursing, Midwifery & Palliative Care, King’s College London, London, UK

**Keywords:** Coping style, Mediating effects, Nurse, Second victim, Professional quality of life

## Abstract

**Background:**

Studies have shown that second-victim experiences could increase risks of the compassion fatigue while support from individuals and organisations is most often protection. However, the risk for poor compassion satisfaction and increased compassion fatigue in nurses aroused by adverse events remains an underestimated problem, meanwhile, litter known about the role of positive and negative coping styles among nurses suffering from adverse events. This study aims to investigate the effect of second-victim experiences on the professional quality of life among nurses and to determine the mediating role of coping styles in the relationship between second-victim experiences and professional quality of life.

**Methods:**

Multistage sampling was used to recruit registered nurses from Hunan province in China. Registered nurses who identified themselves as experiencing adverse events from nine tertiary hospitals were included in this study. Participants were recruited to complete a survey on the second victim experience and support tool, the simplified coping style questionnaire, and the professional quality of life scale. The stress coping theory was used to develop the framework in this study. The structural equation modelling approach was used for conducting the mediating effects analysis via IBM SPSS Statistics 26.0 and Mplus 8.3.

**Results:**

In total, 67% (*n* = 899) of nurses reported a second victim experience during their careers. In a bivariate analysis, both second-victims experiences and coping styles were significantly associated with their professional quality of life. The results showed that the effects of second victim experiences on their professional quality of life were fully mediated by coping styles. A total of 10 significantly indirect pathways were estimated, ranging from -0.243 to 0.173.

**Conclusions:**

Second-victim experiences are common among nurses in this study. Since the mediating effects of coping styles were clarified in this study, it is imperative to promote the perception of negative coping styles and encourage nurses to adopt more positive coping styles with adequate support systems.

## Background

As the largest group of healthcare providers, nurses perform a key role in restoring and promoting health throughout the various life stages and shoulder additional responsibilities such as education and management [1]. Stress is one of the main rationales leading to compassion fatigue among nurses since they are challenged by increasing demands on their expertise and emotions [2]. Notably, providing care in a complex and stressful environment makes nurses more prone to mistakes, which result in a vicious cycle of adverse events [3]. Specifically, among nurses, the strain of maintaining a quality service with few organizational resources and limited appreciation from other health specialists is recognized, and the quality of professional life is of primary concern [4]. Stamm [5] integrated compassion satisfaction and compassion fatigue into the professional quality of life, which refers to the quality individuals perceive and relates to their work as healthcare providers. A systematic review and meta-analysis of professional quality of life among nurses show that the lowest levels of compassion satisfaction and highest levels of compassion fatigue are reported in the Asian region [6]. Compassion fatigue consists of burnout and secondary traumatic stress, which are two different aspects – secondary traumatic stress is less common than burnout; however, it is more powerful in its effect due to its relationship with fear, which is examined as a typical symptom after adverse events [7]. Regarding the impact of adverse events on patients, the risk for poor job satisfaction and reduced professional functioning triggered by adverse events remain an underestimated problem; therefore, the professional quality of life among nurses suffering from adverse events is an imperative issue as it is vital to the delivery and quality of care.

Over 20 years ago, Albert Wu, referred to patients as the first victims and healthcare providers as the second victims of medical errors [8]. The term second victim is defined and extended by Scott [9] and refers to a healthcare provider involved in an unanticipated adverse event that results in psychological and physical effects. Globally, the prevalence of second victim experiences has been reported as ranging from 30 to 60%. Second-victim related stress refers to a series of effects including fear, anxiety, guilt, loss of confidence, flashbacks, rapid heart rate, and sleep disturbances, which are the most common symptoms [10]. Significantly, adverse events related to unsafe care may be one of the leading causes of death and disability in the world [11]. However, nurses may be asked to do at least 100 different tasks while being interrupted at least once every hour, which results in a higher risk of second-victim experiences among nurses than among other professionals [12]. Despite a substantial increase in the prevalence of second-victim experience, there is limited understanding of both second victim experience and its symptoms, and the second victims still face stigmatization of being criticized as unsuitable and inadequate for the job demands. Conversely, second-victim support refers to helping healthcare providers cope with the stress associated with adverse events, which is provided at both individual and organisational levels [13] Since barriers persist in terms of the available assistance, nurses continue to face challenges in obtaining adequate support. This lack of support has been associated with a decline in the quality of care reported [14]. the long-term consequences of second victim-related stress without systematic support pose great risks of family strife, burnout, turnover intentions, and in extreme cases leads to suicide among nurses [15]. Nevertheless, a few studies referring to an association between the components of second-victim experiences and professional quality of life, including positive aspects such as the effects of second-victim support on compassion satisfaction, and negative aspects such as the effects of second-victim related stress, for example, burnout and secondary traumatic stress, which are significant elements for enhancing an understanding response and recognizing the needs of healthcare providers in the aftermath of adverse events.

**Fig. 1:**
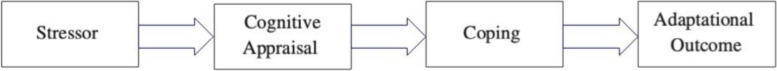
Lazarus and Folkman (1984)’s stress coping model

**Fig. 2:**
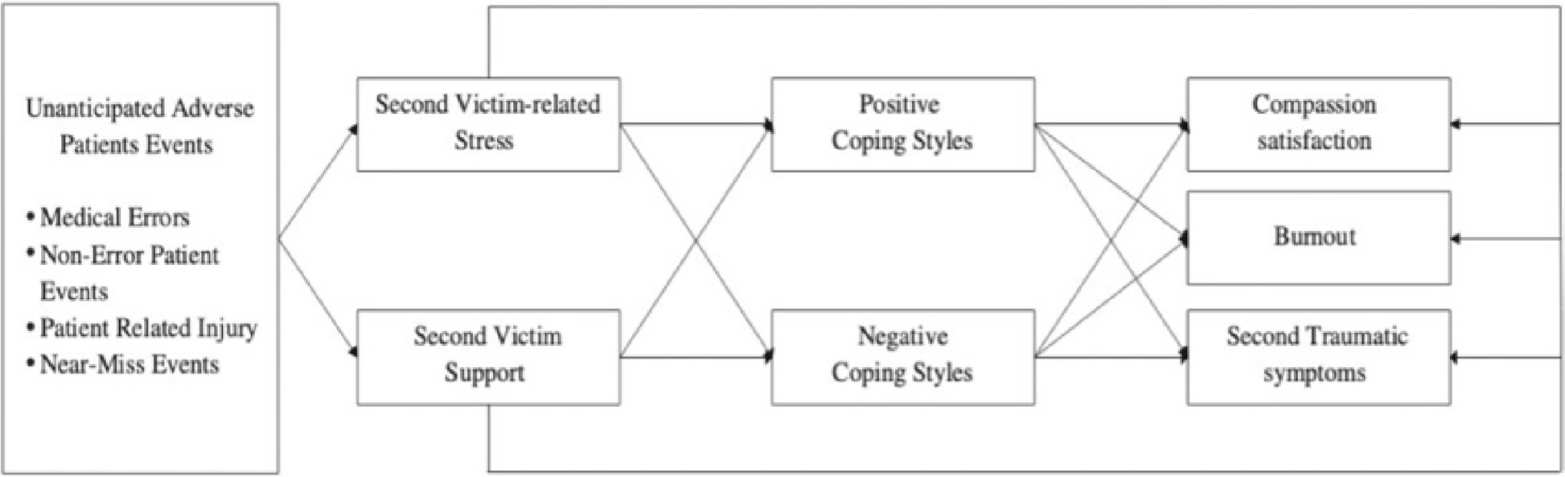
Hypothetical multiple mediation model of coping styles mediating the association among second-victim related stress, second-victim support, and aspects of the professional quality of life

Lazarus [16] defined stress as a relationship between a person and their environment, which is the foundation of the stress coping theory**,** which elucidates the overall coping process, highlighting the sequence of appraisals and subsequent determination of reactions following a stressful situation. The theoretical framework employed in this study is rooted in the stress coping model, which delineates the process of dealing with stressors through cognitive appraisal, coping strategies, and subsequent outcomes **(**Fig. 1**).** Previous research has indicated that coping styles serve as mediator variables in the relationship between stress and distress [17]. To be specific, in the context of second victim experiences, stress situations arise from adverse events. Adverse events refer to injuries resulting in prolonged hospitalization, disability, or even death, which are attributed to healthcare management, including medical errors, non-error events, patient-related injuries, and near-miss events [18, 19]. As cognitive appraisal offering a degree of control over the situation, Yoo [20] mentioned consideration of both the positive and negative dimensions of cognitive appraisal. Healthcare providers who experience adverse events evaluate the negative effects of second-victim related stress and assess the available resources for second-victim support. Based on these evaluations, decisions are made regarding coping styles to be adopted. While coping styles have been found to mediate the relationship between second-victim experiences and nursing practice changes without significant interaction, there is limited information regarding their impact on professional quality of life [21]. Building upon these findings, a framework has been developed to examine the perceived support and coping styles that have the most beneficial effects on stress. In this study, the stressors are represented by adverse events encountered by nurses, such as medical errors and patient-related injuries. Cognitive appraisal pertains to the perceived stress and support that nurses experience during second victimization, assessing their evaluation of these events and the level of support received. The mediating effects of coping styles employed by nurses during second victimization are of central focus. The outcomes are manifested as components of professional quality of life. Ultimately, these variables, through coping styles, contribute to compassion satisfaction, burnout, and secondary traumatic stress. A hypothesized model has been constructed to examine the effects of coping styles in enhancing professional quality of life following second victim experiences **(**Fig. 2**).** This study aims to assess the relationship between the components of second victim experiences and professional quality of life and how it should be mediated by the components of various coping styles.

## Methods

The study followed the STROBE reporting guideline.

### Study design

The descriptive, cross-sectional study was conducted from January to April 2022 in Hunan province, China.

### Setting and participants

The highest number of tertiary hospitals is in the most developed regions of Hunan province with 14. There are 12 hospitals in the second level region and a smaller number of undeveloped regions with 6. The sample size in each region and hospital was determined using proportional-to-size cluster sampling. According to the table of empirical estimates of sample sizes needed for 0.8 power [22], a sample size of 462 or greater is required when using the bias-corrected bootstrapping to test the mediating effect. The inclusion criteria were nurses working as registered nurses for six months or more and experiencing adverse events at least once during their careers. Nurses who were part-time and did not directly deliver care to patients were excluded.

### Procedure

Written informed consent was obtained from the survey participants before the research was conducted. Participants were invited to fill in an online questionnaire via a protected group WeChat and provided with information indicating the purpose, duration, and benefits of the survey, as well as the contact number of researchers.

### Measurements

#### Social demographic characteristics of nurses

The demographic questionnaire was designed to collect personal and occupational details based on findings from previous studies that included gender, marital status, age, education level (from certification to PhD), and department (internal medicine, surgery, etc.).

#### The second victim experience and support tool

The second victim experience and support tool was developed to examine the level of stress and support following an unanticipated adverse event [23]. The Chinese version has 24 items and includes six dimensions, which are psychological distress, physical distress, professional stress, colleague support, manager support, and nonwork-related support. It was measured using a 5-point Likert scale with anchors ranging from 1 (strongly disagree) to 5 (strongly agree), while the dimensions of colleague support, manager support, and nonwork-related support were reversed when scored. High scores indicated a great impact of stress and poor perceived support. Cronbach's alpha of each dimension ranged from 0.71 to 0.90 [24]. In this sample, the Cronbach’s alpha of the tool was 0.85 and Cronbach’s alpha estimates of six dimensions were changed from 0.85 to 0.93. Moreover, the results indicated that the composite reliability of each construct ranged from 0.85 to 0.93(> 0.60), and the average variance extracted of all constructs ranged from 0.59 to 0.85(> 0.50). This suggests that the convergent validity of the second victim experience and support tool in this study was acceptable [25].

#### Simplified coping style questionnaire

The simplified coping style questionnaire is a 20-item self-report scale to assess individual coping styles. It includes a positive coping style (12 items) and a negative coping style (8 items), which were measured using a 4-point Likert scale (0 = never, 1 = seldom, 2 = often, 3 = always). Higher scores of the dimension reflected preferences for the coping style. Cronbach’s alpha for the negative coping style and the positive coping style were 0.89 and 0.78, respectively [26]. Cronbach’s alpha was used to test the reliability in this study (α = 0.89) and Cronbach’s α estimates of two dimensions were 0.92 and 0.82. Moreover, the results indicated that the composite reliability of two constructs were 0.91 and 0.83(> 0.60), and the average variance extracted of two constructs were 0.52 and 0.49, in addition to the factor loadings of the individual items that were all above 0.50. This suggests that convergent validity in this study was acceptable [25].

#### Professional quality of life scale

The professional quality of life scale measures compassion satisfaction, burnout, and secondary traumatic stress among healthcare providers [5]. the Chinese version is a 5-point Likert scale ranging from 1 = Never to 5 = Very often, while items 1, 4, 15, 17, and 29 in the dimension of burnout were reversed when scored, then the items were summed by subscale and the original score was converted to a t‐score (t-score = z-score *10) + 50. A high level referred to a t-score above 57 and below 43 meant a low level. The Cronbach’s alphas of the three subscales were 0.88, 0.81, and 0.75 for compassion satisfaction, burnout, and secondary traumatic stress, respectively [27]. The Cronbach’s alpha was 0.84 in this study and Cronbach’s α estimates of three dimensions were 0.82, 0.81, and 0.80. Moreover, the results indicated that the composite reliability of each construct was 0.84, 0.85, and 0.80 (> 0.60), and the average variance extracted of each construct was 0.58, 0.59 and 0.58 (> 0.50). This suggests that convergent validity in this study was acceptable [25].

### Statistical methods

Data were analysed using IBM SPSS26.0 and Mplus 8.3 [28]. A value of outlier was observed when the standard score (Z-score) was ± 4. The range of Z-score values for all variables was -3.89 to 3.53, indicating that no outliers were observed in the data. The formal normality test (Shapiro–Wilk test) was sensitive to large sample sizes. The absolute value of the skewness was less than 3 and the absolute value of the kurtosis was less than 10, indicating that the data approximately conformed to a normal distribution. A test of common method bias was necessary because a self-reported questionnaire with a multidimensional structure was used to collect data at one point in time. The total variance extracted by one variable was 23.30%, which was less than 50% [29], indicating that the data were unlikely to be affected by the variance of the common method variance. The demographic of participants and their levels of second- victim related stress, second-victim support, coping styles, and professional quality of life were computed by descriptive statistics including frequency, percentage, mean and standard deviation. The relationships among variances were discussed by Pearson's correlation analysis and structural equation modelling was used to analyse the mediating effects of coping styles. Full information maximum likelihood was used to test the hypothesis model and in 1000, bootstrap resamples were run to test the direct and indirect effects [30]. To examine indirect effects of the variable as mediators, compared with confidence interval, it is more recommended set bias-corrected percentile bootstrapping (bootstrap replications:1,000) was performed at a 95% confidence interval [31]. The mediating effects were statistically significant if the 95% bias-corrected bootstrap confidence interval (BCBCI) did not contain 0 [32].

## Results

### Descriptive statistics

A total of 1335 questionnaires were collected and data with incomplete information were deleted, resulting in 1322 valid questionnaires, with a questionnaire validity rate of 99.0%. In total, 899 nurses identified themselves as experiencing adverse events and were asked to provide information on coping styles and professional quality of life. Table 1 presents the demographic characteristics of the nurses (*N* = 899). The data were collected using the following four questionnaires. Table 1 shows the means and standard deviations of demographic characteristics and dimensions, as well as the results of univariate analysis. Female nurses numbered significantly more than male nurses (94.9% vs. 5.1%) and many were married (70.2%, *n* = 165). Approximately 52% (*n* = 470) of nurses were between 30 and 40 years old, while 13.3% (*n* = 120) of nurses were older than 40 years. In terms of working departments, most respondents were from the department of internal medicine, 42% (*n* = 377), followed by 31% (*n* = 279) in the surgery department, with several small departments, paediatrics 14% (*n* = 126) and critical care 13% (*n* = 117). According to the distribution of education level, the highest percentage (87%, *n* = 785) had a bachelor’s degree. Within the sample, 8% (*n* = 69) experienced adverse events within one week, while the largest category consisted of 41% (*n* = 371) who experienced from one year to five years. Additionally, 29% of participants had an experience exceeding five years.

**Table 1:** Demographic characteristics of participants, time of experiencing the adverse events, and dimensions scores (*N* = 899)

Variables/Dimensions	*N* (%)	Mean ± SD
**Gender**		
Male	46 (5%)	
Female	853 (95%)	
**Marital status**		
Single	187 (21%	
Married	691 (77%)	
Divorced	21 (2%)	
**Age**		
≤ 30	309 (34%)	
31–40	470 (52%)	
> 40	120 (13%)	
**Department**		
Internal Medicine	377 (42%)	
Surgery	279 (31%)	
Paediatrics, Obstetrics and Gynaecology	126 (14%)	
Acute and Critical Care	117 (13%)	
**Education level**		
Certification	70 (8%)	
Bachelor	785 (87%)	
Master and above	44 (5%)	
**Time of experiencing the adverse events**		
Within one week	69 (8)	
One week ~ one month	27 (3)	
One month ~ six months	65 (7)	
Six months ~ one years	108 (12)	
One year ~ five years	371 (41)	
More than five years	259 (29)	
**Dimensions scores**		
Second victim related stress		3.43 ± 0.83
R Second victim support		2.13 ± 0.72
Positive coping style		2.01 ± 0.52
Negative coping style		1.45 ± 0.60
Compassion satisfaction		3.41 ± 0.77
Burnout		3.20 ± 0.53
Secondary traumatic stress		2.89 ± 0.71

R second victim support: dimensions have been reversed

No significant associations with professional quality of life were found for gender, age, department, education level, and marital status. As a result, the subsequent structured equation modelling analysis included only second- victim related stress, second-victim support, positive coping styles, negative coping styles, and professional quality of life were included in the subsequent structured equation modelling analysis, excluding demographic data.

In Table 1, second-victim related stress scores and reversed second-victim support scores of (3.43 ± 0.68) and (2.13 ± 0.72), respectively, were measured. The score of the positive coping style (2.01 ± 0.52) was higher than the negative coping style (1.45 ± 0.60). Table 2 shows that nearly 80% of nurses had middle to high levels of compassion satisfaction including middle level, 54.3% (*n* = 488), and high level, 27.4% (*n* = 246), as well as burnout and secondary traumatic stress.

**Table 2:** The score and level of quality of professional life according to domain

	Original score (Mean ± SD)	Level		
		Low (%)	Middle (%)	High (%)
Compassion satisfaction	34.12 ± 7.69	165 (18.4)	488 (54.3)	246 (27.4)
Burnout	32.01 ± 5.31	171 (19.0)	555 (61.7)	173 (19.2)
Secondary traumatic stress	28.88 ± 7.10	197 (21.9)	518 (57.6)	184 (20.5)

### Testing the hypothesis model

**Fig. 3:**
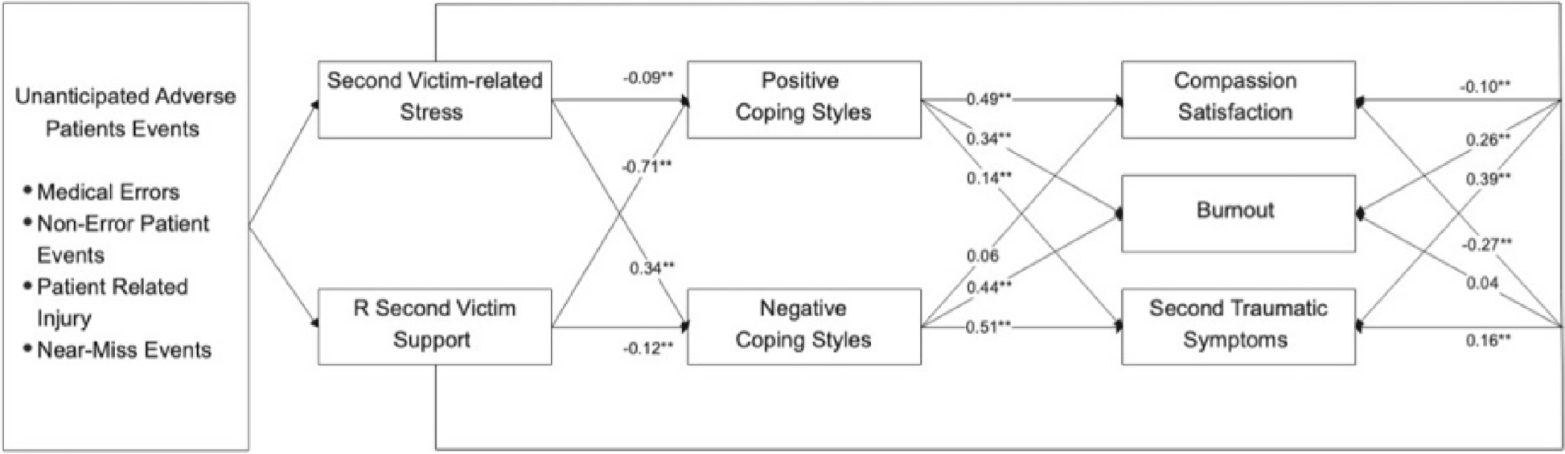
Path model linking second victim experience components and coping styles with the professional quality of life. (R second victim support: items have been reversed). **p < 0.01, *p < 0.05. Model fit: Chi-Square/df = 4.29; RMSEA = 0.06; CFI = 0.90; TLI = 0.90

Table 3 shows most of the correlations of the subconstructs in the measurement model were significant, ranging from 0.104 to 0.555. Fit indices of the final model (Chi-Square/df = 4.29; RMSEA = 0.06; CFI = 0.90; TLI = 0.90) are shown in Fig. 3, which suggests an acceptable fit [33]. The results of path estimates presented in Fig. 3 show that second-victim related stress reduced positive coping styles while it increased negative coping styles. Based on the scoring method of the second victim experience and support tool, the higher score referred to less support, in other words, both the positive coping and negative coping styles were increased by second-victim support. Additionally, three components of professional quality of life were positively affected by positive and negative coping styles.

**Table 3:** The correlations between the measurement model subconstructs

	1	2	3	4	5	6	7
1 Second victim related stress	1						
2 R Second victim support	-0.10**	1					
3 Positive coping style	-0.01	-0.56**	1				
4 Negative coping style	0.33**	-0.13**	0.35**	1			
5 Compassion satisfaction	-0.07*	-0.50**	0.63**	0.18**	1		
6 Burnout	0.34**	-0.24**	0.40**	0.56**	0.53**	1	
7 Secondary traumatic stress	0.47**	-0.06	0.15**	0.56**	0.25**	0.78**	1

R second victim support: dimensions have been reversed

^*^p < 0.05 (two-tailed)

**p < 0.01 (two-tailed)

As shown in Table 4, in the pathway between second victim support and burnout, we found that the total effect is significant, with an estimated indirect effect of -0.257 (95% BCBCI [-0.360, -0.155]), while the direct effect is not significant, with an estimated indirect effect of 0.038 (95% BCBCI [-0.103, 0.164]). However, the indirect effects through positive coping styles and negative coping styles are both significant, with estimated indirect effects of -0.243 (95% BCBCI [-0.340, -0.161]) and -0.052 (95% BCBCI [-0.094, -0.007]). Importantly, the indirect effects of positive coping styles on the relationship between second victim experience and professional quality of life are significant. The second victim experience decreases compassion satisfaction, burnout, and secondary traumatic stress through positive coping styles, with estimated indirect effects of -0.044 (95% BCBCI [-0.084, -0.009]), -0.031 (95% BCBCI [-0.063, -0.005]), -0.012 (95% BCBCI [-0.036, -0.001]), -0.344 (95% BCBCI [-0.438, -0.267]), -0.243 (95% BCBCI [-0.340, -0.161]), and -0.096 (95% BCBCI [-0.188, -0.020]). However, the mediating role of negative coping styles is significant in the dimensions of burnout and secondary traumatic stress on professional quality of life. The second victim-related stress increases burnout and secondary traumatic stress through negative coping styles, with estimated indirect effects of 0.148 (95% BCBCI [0.104, 0.199]) and 0.173 (95% BCBCI [0.126, 0.225]). The second victim support decreases burnout and secondary traumatic stress through negative coping styles, with estimated indirect effects of -0.052 (95% BCBCI [-0.094, -0.007]) and -0.061 (95% BCBCI [-0.109, -0.006]).

**Table 4:** Bootstrap analyses of magnitude and statistical significance of effects

Model pathways	Estimate	95% BCBCI	
		Lower 2.5%	Upper 2.5%
Standardized total effects			
SVRS → CS	-0.126	-0.208	-0.047
SVRS → BO	0.374	0.277	0.472
SVRS → STS	0.551	0.459	0.635
RSVSU → CS	-0.623	-0.698	-0.539
RSVSU → BO	-0.257	-0.360	-0.155
RSVSU → STS	**-0.002**	**-0.102**	**0.096**
Standardized direct effects			
SVRS → CS	-0.101	-0.191	-0.022
SVRS → BO	0.257	0.163	0.344
SVRS → STS	0.39	0.304	0.465
RSVSU → CS	-0.272	-0.405	-0.149
RSVSU → BO	**0.038**	**-0.103**	**0.164**
RSVSU → STS	0.155	0.033	0.289
Standardized indirect effects			
SVRS → PC → CS	-0.044	-0.084	-0.009
SVRS → PC → BO	-0.031	-0.063	-0.005
SVRS → PC → STS	-0.012	-0.036	-0.001
RSVSU → PC → CS	-0.344	-0.438	-0.267
RSVSU → PC → BO	-0.243	-0.340	-0.161
RSVSU → PC → STS	-0.096	-0.188	-0.020
SVRS → NC → CS	**0.019**	**-0.005**	**0.051**
SVRS → NC → BO	0.148	0.104	0.199
SVRS → NC → STS	0.173	0.126	0.225
RSVSU → NC → CS	**-0.007**	**-0.023**	**0.001**
RSVSU → NC → BO	-0.052	-0.094	-0.007
RSVSU → NC → STS	-0.061	-0.109	-0.006

Standardized estimating of 1,000 bootstrap resample

Bold text refers to not statistically significant when the 95% BCBCI contains 0

*SVRS* second victim-related stress, *RSVSU* reversed second victim support, *PC* positive coping styles; *NC* negative coping style, *CS* compassion satisfaction, *BO* burnout, *STS* secondary traumatic stress, *BCBCI* bias-corrected bootstrap confidence interval

## Discussion

Findings in this study provided initial evidence of how components of second victim experience affect the professional quality of life among nurses. Second-victim related stress may lead to a significant increase in symptoms of burnout and secondary traumatic stress, while compassion satisfaction decreases by second-victim related stress, which is identified in this study. In line with previous findings, the second victim experiences result in anxiety, fear, exhaustion, and loss of hope [34]. Therefore, the arousal caused by second-victim experiences following crisis events, along with the everyday pressure experienced by nurses can lead to poor compassion satisfaction [35]. Moreover, exposures to adverse events increase the consumption of psychological and physical energy, so nurses experience aggravated mental and emotional fatigue leading to burnout. Notably, in comparison to burnout, secondary traumatic stress is aroused by adverse events among nurses, resulting in more risks for patient safety, which creates a malicious cycle and may give rise to suicidal ideation [36].

In terms of second victim support, this is found to be an immediate protective factor of professional quality of life in this study, reducing the symptoms of compassion fatigue and promoting compassion satisfaction. In addition, poor manager support is a significant predictor of lower compassion satisfaction and higher levels of compassion fatigue among nurses, while improved colleague relationships positively affect compassion satisfaction, This is evidence that intervention to prevent nurses being blamed and punished for adverse events is the key for professional quality of life and quality of care [37]. However, many nurses struggle with the shame and stigma associated with adverse events, caused by a lack of financial support, lack of system support, and possibly by a culture that is not well established [38]. Once the exhaustion stage sets in, psychological stress, physical stress and professional stress result in a loss of confidence, and turnover intention [39]. Therefore, in the aftermath of adverse events, organizations and managers need to recognize the costs of fatigue and take measures to improve the environment and develop support systems for recovery.

Results in this study broaden the understanding of the effect of coping styles. In second- victim experience samples, this study finds that the positive coping style is a significant mediator in compassion satisfaction, not only reducing the negative effects of second- victim related stress but also enhancing protection based on second-victim support for compassion satisfaction. These results corroborate findings in China that positive coping styles could assist in perceiving and buffering the negative effects of second victim symptoms, thus enhancing optimistic emotions, which may increase job satisfaction, employee retention rate and well-being [40].

An important finding from this study is that positive coping styles may lead to an active, although indirect, effect on burnout and secondary traumatic stress in the aftermath of adverse events, which means positive coping styles may lead to struggling situations. It supports the claim that Chinese nurses fear disclosure errors and even near-miss events and tend to manage their sadness, depression, and anxiety by themselves [41]. Additionally, this study demonstrated significant indirect effects of positive coping styles between perceived support and secondary traumatic stress. Although no direct effects on burnout could be established, positive coping styles showed complete mediation in the relationship between second victim support and burnout. in other words, certain styles of positive coping styles, if conducted in a poor team environment, are not effective in reducing stress and may even result in worsening the situation [42]. Overall, support and positive coping styles contribute to the promotion of compassion satisfaction among nurses involved in adverse events, however, it is critical to note that a blindingly emphasis on positive coping styles and responsibility may be counterproductive in an inadequate organizational culture and environment.

On the other hand, negative coping styles primarily act as mediators between second- victim experiences and compassion fatigue, referring to dealing with second-victim experiences in a negative coping style, which may worsen risks of both burnout and secondary traumatic stress. This pattern of negative coping is in line with a previous study that reported that although the negative coping style can alleviate symptoms in the short period, it can be detrimental in the long term, for example, adopting unhealthy coping styles such as drinking and smoking or choosing an escape route and refusing individuals or organizations support for some time, which may result in further increased risk of compassion fatigue instead of overcoming the negative effects from adverse events [1].

### Strengths

A reliable analysis was carried out via a structural equation model by Mplus and the sample size, in region and hospital with different levels of economic development, was determined using proportional-to-size cluster sampling. This study found a robust relationship among second victim experiences, coping styles, and professional quality of life. Foremost, second-victim experiences were found to be detrimental to professional quality of life, including reduced compassion satisfaction and increased risk of burnout and secondary traumatic stress. Secondly, in terms of coping styles, positive coping overall showed to be a protective mediator, but in an inadequate organizational culture and environment, positive coping by nurses may still aggravate the risk of burnout and secondary traumatic stress. On the other hand, negative coping not only worsens the passive effects of second-victim related stress but also weakens the active effects of second-victim support.

### Limitations

This study is a cross-sectional survey conducted through self-reporting questionnaires, therefore, deviations in reporting are inevitable and further studies need to consider the longitudinal research and explore the effects of coping styles between second victim experience and patient outcomes. Additionally, the zero-tolerance policy on adverse events and lack of understanding about second-victim experiences may result in no report among nurses. Furthermore, the results reflected the situation in tertiary hospitals only, and a widely survey of different hospital levels is required to extend understanding in this field of research and mixed methods research must be combined.

## Conclusion

Second-victim experiences are common among nurses in this study. Nowadays, in a technological and complex healthcare environment, it is impractical and impossible to ignore the increasing compassion fatigue and decreasing compassion satisfaction experienced by nurses who have suffered from adverse events. In this study, the mediating effects of coping styles were established, indicating their importance in understanding the relationship between second victim experiences and professional quality of life. By promoting the perception of negative coping styles, particularly their mediating role in exacerbating burnout and secondary traumatic stress, nurses can become more aware of the potential negative consequences of negative coping styles aftermath of second-victim experiences. This awareness can serve as a start for change, encouraging nurses to adopt positive coping styles. Specifically, this study highlighted that excessive emphasis on positive coping styles related to potential risks and may lead to harm to the compassion satisfaction in professional quality of life in situations where support systems are inadequate. Additionally, it underscores the importance of promoting positive coping styles to mitigate the harmful effects of second-victim experiences, promote support perceived, and enhance professional quality of life.

## Implications for the profession

This study demonstrated a higher occurrence of compassion fatigue than previous studies [6]. It is noteworthy that necessary to promote the understanding of second-victim experiences and control negative coping styles. Hospitals and nurse leaders need to provide training programs for nurses prior to support programs, which are intended to enhance a comprehensive understanding of the second-victim experience and to promote perceiving the disadvantages of negative coping styles to change the positive styles, as well as to highlight the awareness of help-seeking resulting in positive coping styles.

## Data Availability

The datasets generated during and/or analysed during the current study will be shared only on request with the approval from the Universiti Malaya thesis Unit. Please contacted corresponding author Mei Chan Chong mcchong@um.edu.my upon reasonable request.
